# Floating-Gate MOS Transistor with Dynamic Biasing as a Radiation Sensor

**DOI:** 10.3390/s20113329

**Published:** 2020-06-11

**Authors:** Stefan Ilić, Aleksandar Jevtić, Srboljub Stanković, Goran Ristić

**Affiliations:** 1 Applied Physics Laboratory, Faculty of Electronic Engineering, University of Niš, 18000 Niš, Serbia; ilic.stefan@elfak.rs (S.I.); aleksandar.jevtic@elfak.rs (A.J.); 2 Department of Radiation and Environmental Protection, “Vinča” Institute of Nuclear Sciences, 11351 Belgrade, Serbia; srbas@vin.bg.ac.rs

**Keywords:** floating-gate MOS transistor, ionizing radiation sensor, ZTC, semiconductor dosimeter, transistor dynamic biasing

## Abstract

This paper describes the possibility of using an Electrically Programmable Analog Device (EPAD) as a gamma radiation sensor. Zero-biased EPAD has the lowest fading and the highest sensitivity in the 300 Gy dose range. Dynamic bias of the control gate during irradiation was presented for the first time; this method achieved higher sensitivity compared to static-biased EPADs and better linear dependence. Due to the degradation of the transfer characteristics of EPAD during irradiation, a function of the safe operation area has been found that determines the maximum voltage at the control gate for the desired dose, which will not lead to degradation of the transistor. Using an energy band diagram, it was explained why the zero-biased EPAD has higher sensitivity than the static-biased EPAD.

## 1. Introduction

Further technology development of civilization, such as higher demands for energy, space explorations, accelerator experiments, puts us in increasing contact with ionizing radiation. To monitor and early detect higher radiation level, there is a growing need for ionizing radiation sensors. Therefore, the radiation sensor should be miniature, low power, compatible with other electronics in the system, etc., so that it can be used in variable situations. All these needs can best meet the semiconductor radiation dosimeters.

Various semiconductor components are widely used as a radiation sensors and have many applications, such as PIN diodes [1], PIN photodiodes [2], phototransistors [3,4], Light-Dependent Resistors (LDR) [5], PMOS [6,7,8] and VDMOS transistors [9,10].

The first paper describing that Metal-Oxide-Semiconductor (MOS) transistor can be used as a radiation dosimeter is written by Holmes-Siedle et al. [11], who is considered to be a pioneer in this field. When MOS transistor is irradiated it collects the positive charge in the silicon dioxide. Thus, threshold voltage is shifted, which is proportional to the absorbed dose. Hughes et al. [12] showed that dual dielectric MOS transistor as a radiation sensor increase stability compared with conventional MOS transistor. Silicon nitride layer was deposit on top of high-quality thermal silicon dioxide. When negative gate bias was applied during irradiation, the holes are trapped near or at the $SiN-SiO_{2}$ interface. It was leading to the use of a floating-gate MOS transistor as a radiation dosimeter that was first presented by Kassabov et al. [13] and Snyder et al. [14]. The floating gate is a polycrystalline structure that stores the charge which eliminates the instabilities that occur in the oxide by irradiation. Although there is a hybrid with an ion chamber and a floating gate, called Direct Ion Storage (DIS) [15,16]. They are widely used because of the high sensitivity and low fading. However, DIS dosimeters are not CMOS compatible, which makes them not suitable for monolithic integration. Therefore, a well-designed floating-gate dosimeter can be competent with other sensors on the market.

**Figure 1:**
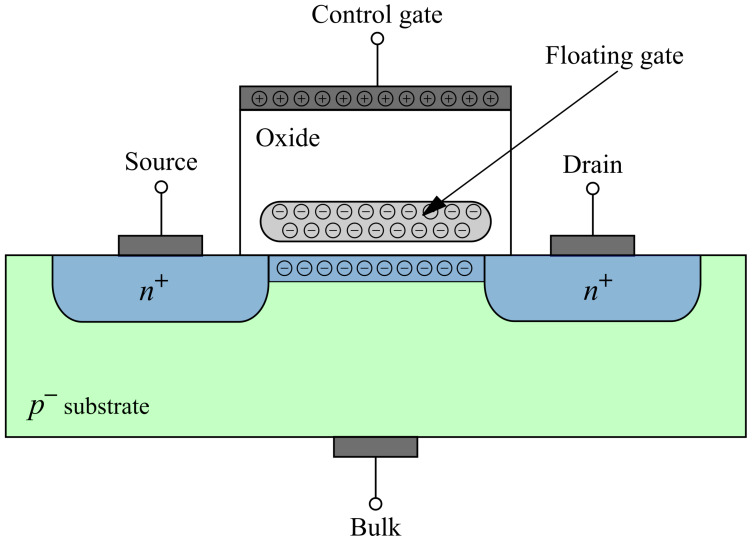
MOS transistor with the charged floating gate.

Floating-gate MOS transistors are at the core of every modern nonvolatile memories [17]. A floating-gate MOS structure consists of two gates: floating and control gate, as shown in Figure 1. A floating gate is surrounded by silicon dioxide to be isolated from the rest of the MOS structure, and it is usually made of polysilicon. The control gate is a gate on ordinary MOS transistor. To charge the floating gate, a positive bias should be applied on the control gate to drag the electrons from the channel to the floating gate (FG). Of course, the channel needs to be formed first by putting a potential between the source and the drain.

**Figure 2:**
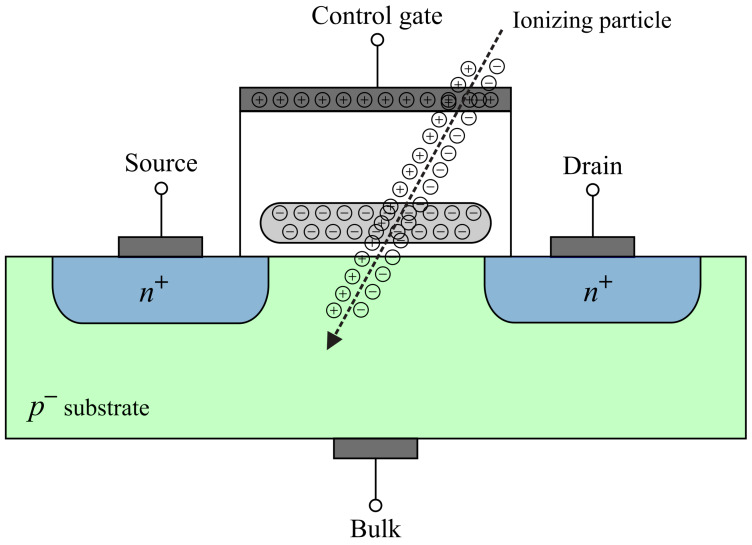
Floating-gate MOS transistor during irradiation.

When an ionizing particle hits the MOS transistor with a floating gate, it generates the electron-hole pairs (Figure 2). The electric field from the floating gate can separate holes and electrons. There are three mechanisms which could cause a threshold voltage decrease: holes injected into the FG, holes trapped in the oxide and electron emission over the polysilicon/oxide barriers [14]. The external electric field from the control gate can help internal field (from the FG) in the separation process of generated charge. There is one more mechanism that is unavoidable in all MOS transistors during irradiation, and that is a degradation of structure between the oxide and the channel (bulk), inducing the generation of an interface (switching) traps [18]. Interface traps can also shift the threshold voltage, and they are responsible for the higher drift of threshold voltage [19].

An ideal floating-gate MOS dosimeter (FGDOS) will be now described. Two most important characteristics of any dosimeter are sensitivity and stability (over time) [20]. The active material of FGDOS is a floating gate. To increase sensitivity, the floating gate should be as large as possible. Of course, there are limitations: charging of the FG will be prolonged, dosimeter will occupy significant space on the chip, there is a potential problem with charge retention, etc. Perhaps, if the floating gate is massive, charging the FG should be achieved at more than one points, which will also complicate the design and the charging procedure. During irradiation, ideal FGDOS should only have a change of the FG charge. Interface traps and fixed traps in the oxide far from the FG are undesirable because they will make dosimeter unstable during the time. A shift of the threshold voltage after irradiation caused by physical processes inside the device is called fading, and it is known as a loss of the information. Therefore, fading should be as minimum as possible, and that can be achieved if the decrease of the FG charge is dominant over all other (undesirable) mechanisms in the device during irradiation. Also, charge retention is an essential parameter of the FG structure; for nonvolatile memories, typical retention time is ten years [21]. Therefore, ideal FGDOS should keep the charge of the FG during a long time at a wide range of ambient temperatures. The ideal FGDOS should be reusable. After discharging of the FG, an FGDOS should be capable of charging again to the same initial value (fully charged) and with the same dosimetric characteristics as it was before.

### Electrically Programmable Analog Device

**Figure 3:**
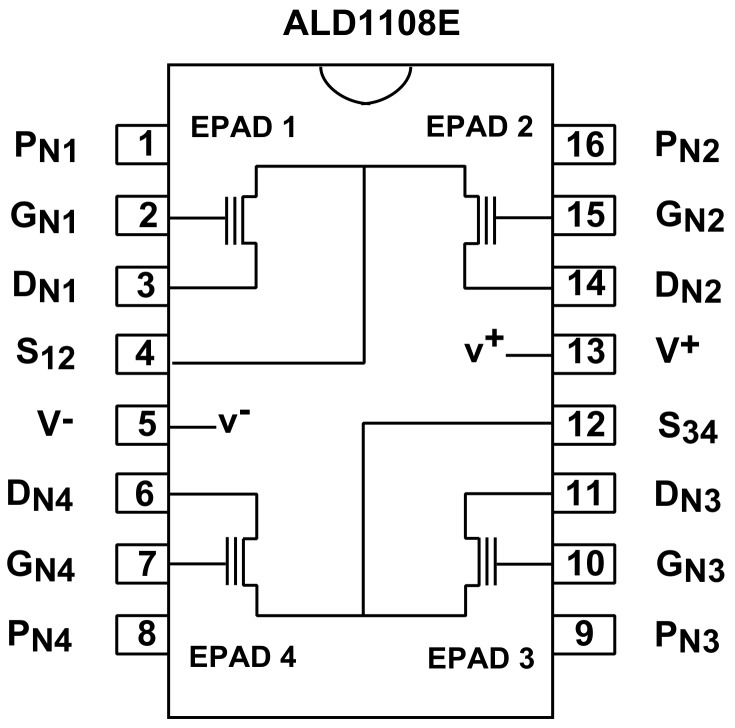
Pinout of the integrated circuit ALD1108E [22].

EPAD (Electrically Programmable Analog Device) is N-channel MOSFET with electrically adjustable threshold voltage, suitable for matched-pair balanced circuit configurations, such as current sources and current mirrors [22]. It is very rare on the market, but it has a lot of advantages due to its floating-gate MOS structure [23]. In this paper, we used commercial IC ALD1108E, which consists of four EPADs manufactured by (Advanced Linear Devices, Sunnyvale, California, USA). Each EPAD has a programming pin (Figure 3). Programming means precisely controlling the threshold voltage by storing the nonvolatile charge in the floating gate of the transistor. Without the possibility of buying a programmer, the authors designed their programmer which was calibrated and tested [24]. That programmer can precisely adjust the threshold voltage from 1 V to 4 V.

This paper aims to analyze the feasibility of floating-gate MOS transistor, such as EPAD, for use in radiation sensors. The idea of using EPAD as an radiation dosimeter was first introduced in Edgecock et al. [25] in 2009., and in 2012., a doctoral dissertation on the same topic was published by the same group of authors [26]. However, no scientific papers on EPADs except our [27] have been published to date. The aforementioned group of authors irradiated EPADs to an absorbed dose of about 100 Gy, and the initial threshold voltage varied from the parameters. Because they used a commercial programmer, the maximum threshold voltage recorded was 3.5 V. Our experiment is planned to go to higher doses, and the programmer described in our paper [24] can charge EPADs up to and beyond 4 V. The highest stable voltage we reached was 4.5 V, but due to the very long programming time, values after 4 V were not considered for this experiment.

## 2. Materials and Methods

In this section, first the structure of EPAD will be analyzed. A dynamic bias will be explained, after that an automated system for measurements of EPADs will be presented, and at the end of section experimental setup during irradiation will be shown.

### 2.1. Reverse Engineering of Epad

**Figure 4:**
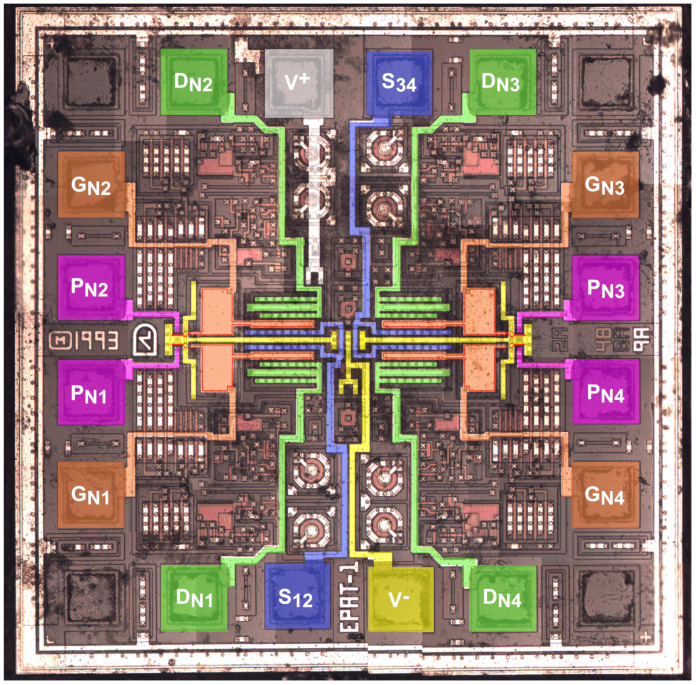
Illustrative composite image of ALD1108E under an optical microscope, edited by Inkscape [28].

To understand the floating-gate MOS structure of EPAD and further explain the behavior of these transistors under the effects of radiation, we decapsulated and then analyzed the ALD1108E IC using an optical microscope [28]. Figure 4 shows a composite image of ALD1108E under an optical microscope, edited using Inkscape. By analyzing this image, we can notice four symmetrically distributed NMOS transistors in the center of the chip.

**Figure 5:**
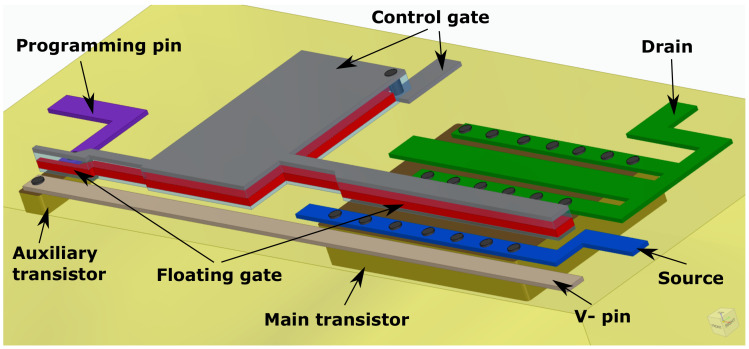
A simplified 3D representation of the EPAD structure.

To further understand the structure of the EPAD, we create a simplified 3D representation of a single transistor from this chip using MCAD Solid Edge Academic. Figure 5 shows the isometric view of a simplified 3D representation of the structure of the EPAD.

**Figure 6:**
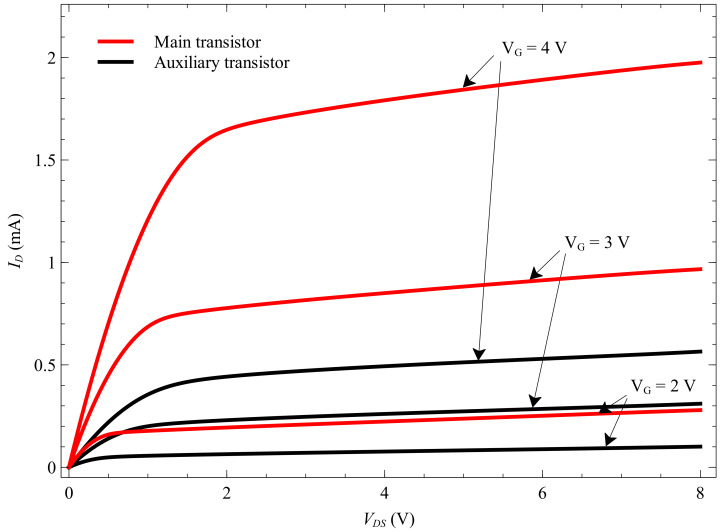
Output characteristics of the main and auxiliary transistor.

We can distinguish between the main and auxiliary transistor. The programming pin is the drain terminal, whereas the V-pin is the source for the auxiliary transistor. To confirm that assumption, we measured the output characteristics of the main and auxiliary transistor (Figure 6). The control gate (grey layer) and a floating gate (red layer) are common for both transistors. We assumed that there is a big area of floating and control gate layers between the auxiliary and main transistor. That area is the storage for electrons so the EPAD can achieve great stability [22]. The purpose of the auxiliary transistor is to charge the floating gate by injecting hot carriers near the drain or by tunneling [25]. A very similar structure is represented by Tarr et al. [29]. Their design has no auxiliary transistor, only an injector gate with the same purpose.

Comparing the output characteristics of the main and auxiliary transistor we can assume that the main transistor has a larger area on the chip, which is confirmed by the composite image of the ALD1108E IC shown in Figure 4.

**Figure 7:**
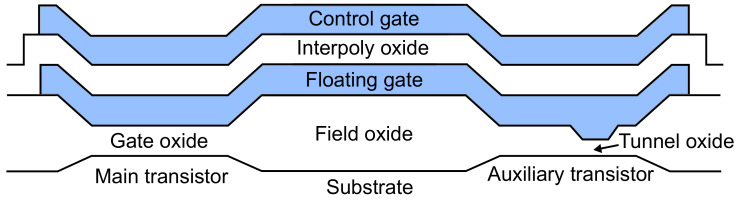
Illustration of the EPAD structure cross-section.

Based on everything previously explained, we drew the cross-section of the EPAD structure (Figure 7). Floating gate and control gate are extending along the entire cross-section. Interpoly oxide is isolating the two gates; it is usually made of oxide-nitride-oxide (ONO) layer. Field oxide is very thick silicon-dioxide layer; its purpose is to minimize the effects of the electric field from the floating gate on the substrate and to isolate the large area of the floating gate. Although field oxide is essential for using this structure as a floating-gate dosimeter because we need large volume for the radiation-generated charge (electron-hole pairs), and field oxide is perfect for that purpose [30]. Tunnel oxide is very thin silicon-dioxide layer, it is used for charging the floating gate, and it degrades with charging cycles. As mentioned above, the auxiliary transistor is designed for charging the floating gate. Therefore, degradation of oxide structure because of FG charging that is inevitable is not affecting the main transistor. During irradiation, all oxides in the structure have electron-hole pairs generated. Amount of radiation-generated charge depends on the electric field in the oxide and the oxide thickness [31]. Therefore, field oxide will produce the most electron-hole pairs, but it will also be the most degraded oxide, which is good because degradation from the field oxide and tunnel oxide will not affect the main transistor. Here, we can conclude that this structure can provide stability for the dosimeter application. Considering that EPAD is fabricated in CMOS technology, a future dosimeter based on this structure can be monolithic.

### 2.2. Dynamic Biasing

A new idea in this experiment that has not been seen in the literature so far is the dynamic biasing of the MOS transistor gate during irradiation. Specifically, a floating-gate MOS transistor has two electric fields, one internal, which originates from the charge at the floating gate, and the other, external, which originates from the control gate. Due to irradiation the charge on the floating gate decreases, and the internal electric field is weakened and, consequently, a smaller amount of generated electron-hole pairs in the oxide occurs. This leads to a slighter decrease of the FG charge, smaller shift in threshold voltage during irradiation, and less sensitivity of EPAD to ionizing radiation.

The idea of the dynamic bias is to amplify the external electric field during the irradiation, due to the weakening of the internal field, and thus maintain an approximately constant electric field value in the oxide, i.e., constant sensitivity of EPAD. The goal of dynamic bias is to achieve a linear dependence of the threshold voltage change on the dose received, to simplify the calibration of EPAD as an ionizing radiation sensor.

**Figure 8:**
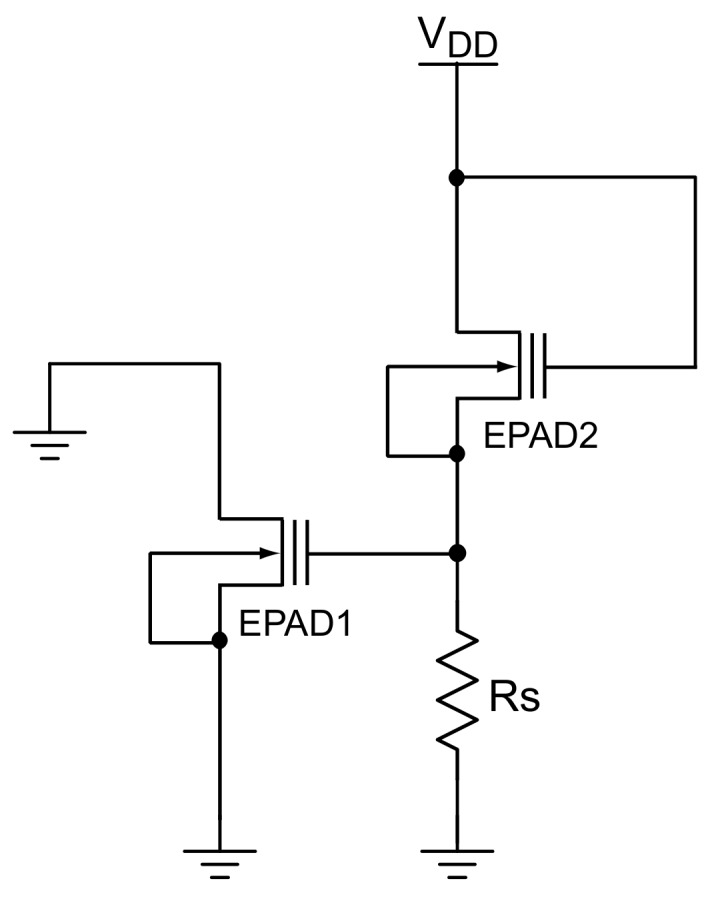
Idea of self-compensated dynamic biasing.

To achieve dynamic bias, it is necessary to achieve such change in a bias at the control gate, which will monitor the effect of ionizing radiation. When irradiation is interrupted, EPAD must retain the last voltage value on the control gate until radiation continues. Such a requirement excludes the dynamic biasing that would come from a signal generator or other programmable controlled power source; it must be rather a self-compensated system. This led us to design a system of two EPADs, as shown in Figure 8.

Therefore, EPAD1 will be a sensor whose threshold voltage is monitored during irradiation, while EPAD2 will be in a voltage divider with a constant resistor. During irradiation, the voltage value on EPAD2 decrease, while the voltage value on the resistor increase, and therefore the control gate of the first EPAD increase.

### 2.3. Automated System for Measurements

Automatic measurement gives great advantages over manual measurement. It takes much time in the design process of the automation system, but the time spent can pay off many times over. By manual measurement, it would be necessary to interrupt the experiment, remove the components from the board and measure the transistor characteristics. Each interruption of the experiment, removing and reassembling the components in front of the radiation source, enters additional time and increases the likelihood of error during reassembly, especially if the experiment is very long and demanding. The switching matrix can give a solution to the problem posed. However, the number of outputs the switching matrix has limits the capabilities and capacity of the experiment. It is also necessary to install many cables from the switching matrix to the component board which makes mounting difficult. It can also produce more noise because of the long cables as the relays are switching in the matrix rather than directly on the board, near the components.

To avoid the problems mentioned above, an automated measuring system was designed for EPAD purposes. The system is universal and can be used for other components or experiments, which was also one of the goals when designing this system.

The system consists of a printed circuit board with relays and EPADs (mainboard), Switching and Bias Unit (SABU), a dual-channel SMU (2636A Source Meter, Keithley Instruments, Solon, OH, USA) and a computer that controls the entire system using a Windows application written in C#.

**Figure 9:**
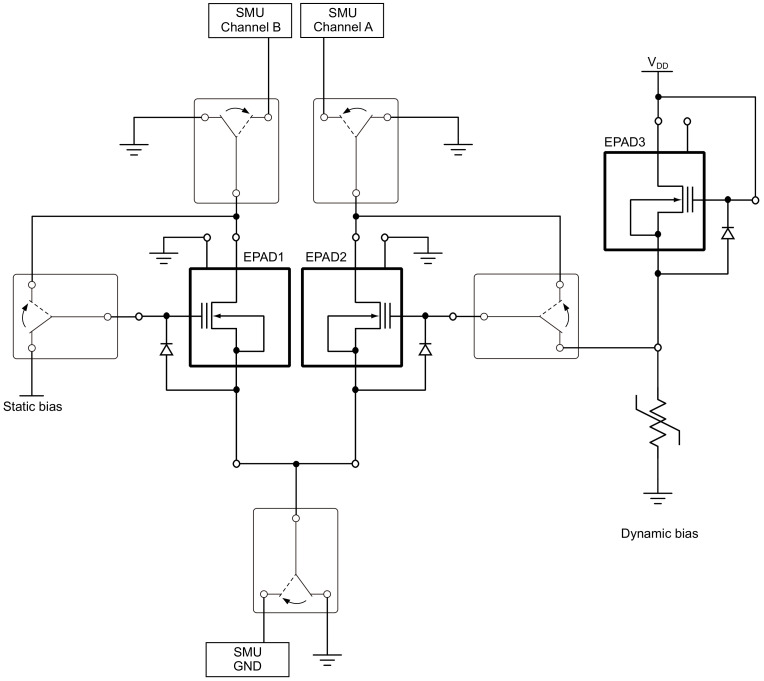
Electrical schematic of EPADs block.

The printed circuit board with EPADs (mainboard) consists of eight identical blocks, and each block contains one ALD1108E integrated circuit with four monolithic integrated EPADs, five relays and one trimmer potentiometer. The electrical schematic of one block is shown in Figure 9. Of the four transistors on the ALD1108E chip, one was not used. That transistor will also be irradiated during the experiment, but it will not be measured and analyzed.

As can be seen in Figure 9, there are two configurations. One, when the relays are in the neutral position, to bias the transistors during irradiation steps, and the other, when all relays are active, to measure the I-V characteristics of EPADs. Neutral position of the dynamic bias corresponds the electrical schematic shown in Figure 8. EPAD1 and EPAD2 are intended for measurement, while EPAD3 provides dynamic biasing for EPAD2. It should be emphasized that the ground of the biasing part and dual-channel SMU is separated to minimize the noise during the measurement. Since all relays need to be switched on simultaneously in one block, it is possible to design a system with double relays (two relays, one coil), which reduces the number of pins for relay control. It may be noted that the programming pin and V-pin are grounded since the auxiliary transistors were not part of the study of this experiment.

The Switching And Bias Unit (SABU) is an auxiliary printed circuit board for control of relays and providing different voltages for biasing the components during irradiation. The board can control 24 relays and bias 8 different voltages values. It contains a PIC16F887 microcontroller, which communicates with the computer via the FTDI chip. A 5 V power supply for the microcontroller is obtained from the computer, while the power for relay switching (12 V) and component biasing (15 V) is provided from two linear power regulators. An ICSP socket is designed to directly program the microcontroller on the board using a PIC programmer. The microcontroller controls relays via the optocouplers. To activate the desired relay, it is necessary to send a specific character from the computer via the FTDI chip. By sending another character, a specific relay goes down.

It should be noted that it was possible to design the entire system on a single PCB, but then the secondary electronics serving the EPADs would be exposed to radiation, which would drastically reduce the reliability of such a system. Also, since the SABU is independent and universal, it can serve for other experiments. Even if the experiment requirements exceed the capacity provided by a single SABU board, it is possible to pair multiple such boards, which will be independently controlled via a computer.

In Appendix B, the photos of Switching And Bias Unit Figure A3, top view of the mainboard with relays Figure A4 and bottom view of the mainboard with irradiated EPADs Figure A5 are shown.

### 2.4. Experimental Setup

As already mentioned, two printed circuit boards were designed, the mainboard with EPADs, which was exposed to radiation during the experiment, and the auxiliary control panel, the so-called. SABU, which serves the mainboard. The arrangement of the EPADs on the mainboard is radial, the center of the circle described by the EPADs aligned with the center of the ionizing radiation field so that all components receive the same dose. EPADs are located on the bottom of the PCB, while all other components are on the top of PCB. The mainboard with EPADs is mounted on a 3 mm thick PMMA plexiglass, using a spacer so that the EPAD housings adhere to the plexiglass (standard for gamma-ray irradiation), while the plexiglass is fixed to the collimator mouth.

**Figure 10:**
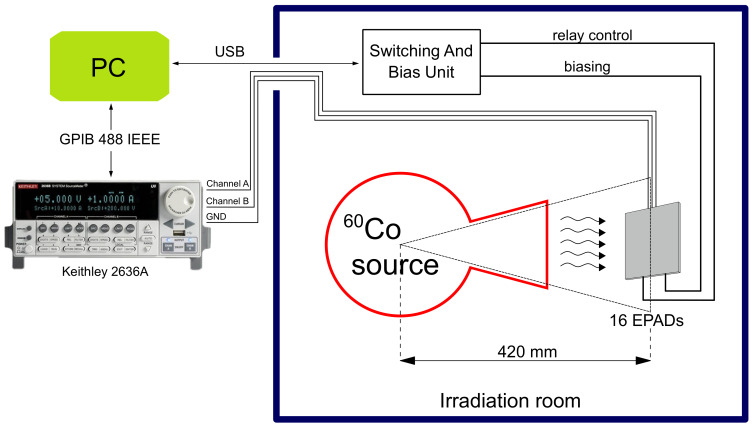
Experimental setup in irradiation room at Institute of Nuclear Sciences “Vinča”.

The experimental setup consists of 16 EPADs (8 ALD1108E integrated circuits) whose transfer characteristics are measured cyclically throughout the experiment (irradiation steps). The experiment consisted of two phases, irradiation and spontaneous recovery. Irradiation was performed to a total absorbed dose of 750 Gy, at a dose rate of 32.645 Gy/h in water, and spontaneous recovery took place at 27 ${}^{\circ }C$ and lasted for a total of 46 days (1107.7 h). The setting of the experiment during irradiation is presented in Figure 10.

The irradiation was performed with a cobalt radioactive isotope ${}^{60}Co$. The components were 420 mm from the source, because it is the closest point to the radiation source (collimator mouth). In the irradiation room, SABU and power supplies are obscured by leaded panels to protect from the reflected gamma radiation. Only a mainboard with EPADs was directly exposed in the radiation field that consists of a high-energy photons beam.

The first part of the experiment took place in the Laboratory of Radiation and Environmental Protection at the “Vinča” Institute of Nuclear Sciences, Belgrade, Serbia, in July 2019. The second part was done at the Applied Physics Laboratory at the Faculty of Electronic Engineering, University of Niš, Serbia.

## 3. Results

All transfer characteristics of EPADs are measured with Keithley 2636 SMU, forcing the current and measuring the voltage. Drain and gate are shorted during measurement and configuration is known as a reader circuit [32]. To use MOS transistor as a radiation sensor, we need to monitor the shift of the threshold voltage; in practice, that means forcing the constant current in reader-circuit configuration and measuring the voltage shift.

**Figure 11:**
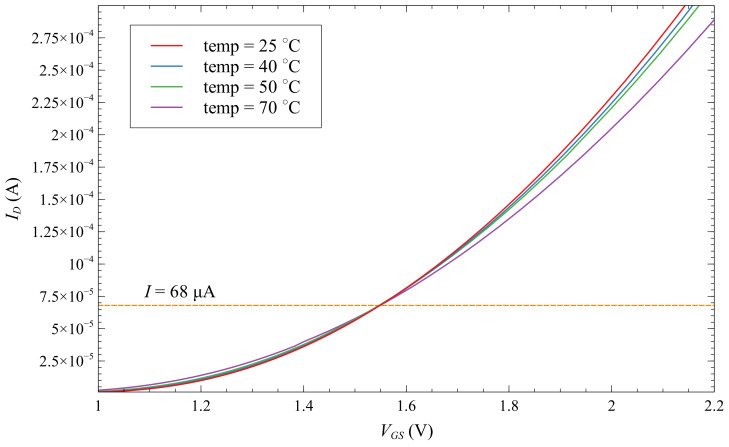
Transfer characteristics of EPADs for different ambient temperatures—Determination of Zero Temperature Coefficient point.

The EPAD threshold voltage is defined, by the manufacturer, as the value of the voltage at current $I_{D}=1\;\mu A$ [22]. Zero temperature coefficient (ZTC) point is defined for current value of $I_{D}=68\;\mu A$. As the value of ZTC current is a very important parameter for the EPAD operation as a radiation sensor, the manufacturer’s claim was experimentally confirmed, and the graph is shown in the Figure 11. We can observe the overlap at a current value of 68μA of the transfer characteristics of EPAD measured at different temperatures. The threshold voltage of the measured EPAD was $V_{th}=1\;V$. Using reader-circuit configuration in ZTC point can provide temperature independence of the radiation sensor. Thus, ZTC point is a significant parameter.

There are two big groups of EPADs in this experiment. The first group had a static bias, and second group had a dynamic bias during irradiation.

### 3.1. Static Bias

Group of EPADs with static bias during the first phase of the experiment, consist of 8 EPADs, and have two subgroups: zero bias and static bias (higher values than zero). Their parameters such as bias during irradiation and initial threshold voltage are represented in Table 1.

A subgroup of static-biased EPADs all have an initial threshold voltage of 4 V, and they are biased with values: 2.5, 5, 7.5 and 10 V during irradiation. Zero-biased subgroup of EPADs have initial threshold voltage values: 1, 2, 3 and 4 volts, their control gates are grounded during the experiment.

**Figure 12:**
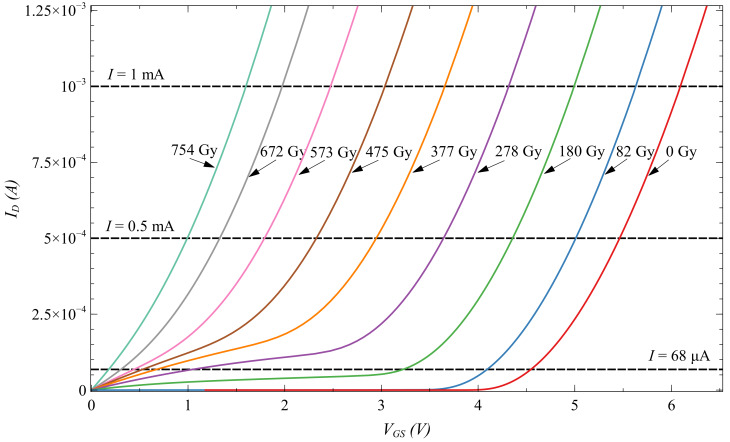
Degradation of transfer characteristic of EPAD with 10 V bias during irradiation.

Degradation of EPAD transfer characteristic increases with the absorbed dose, and with higher bias value. We can observe in Figure 12 the degradation of the transfer characteristic (EPAD8) with the absorbed dose. Characteristic is shifting from the right to the left because the floating gate is discharging, and thus threshold voltage is decreasing. As can be seen, the degradation is in the lower part of the transfer characteristic, and it is increasing during irradiation. Therefore, some parts of the characteristic cannot be read anymore. Threshold voltage can be read until the 82 Gy, and ZTC until the 180 Gy for the 10 V bias.

**Figure 13:**
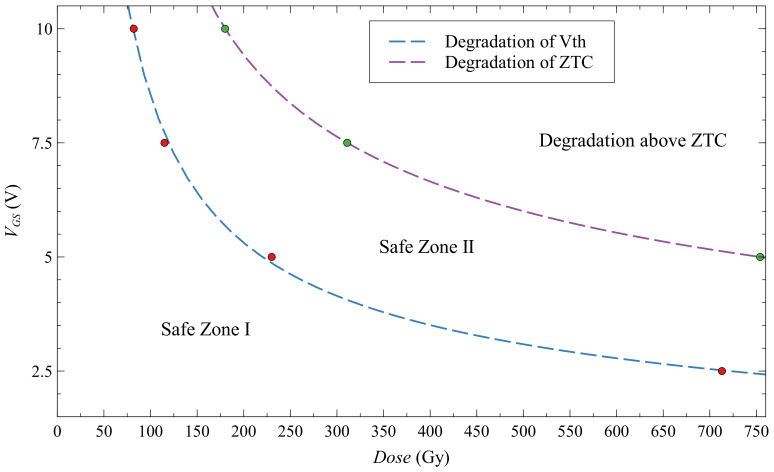
Safe Operating Area of EPAD. Red dots are representing the measured values for Vth ($I_{D}=1\;\mu A$) and green dots for ZTC ($I_{D}=68\;\mu A$) values.

Because of this problem with degradation, we decided to make a Safe Operating Area of EPAD based on all information from the group of EPADs with static bias (Figure 13). Safe Zone 1 is the area where the threshold voltage can be monitored, and Safe Zone 2 is the area where ZTC voltage can be monitored. Above curve that is representing the degradation of ZTC, it can be traced only higher than ZTC values. EPADs with zero bias are not degraded, and curves that are describing the degradation of Vth and ZTC are only converging to zero. Degradation of transfer characteristic of EPADs can be explained as a parallel resistive path that was opened in the floating-gate MOS structure [27]. In our earlier work, we showed that after annealing at 70 ${}^{\circ }C$ transfer characteristics had recovered.

**Figure A1:**
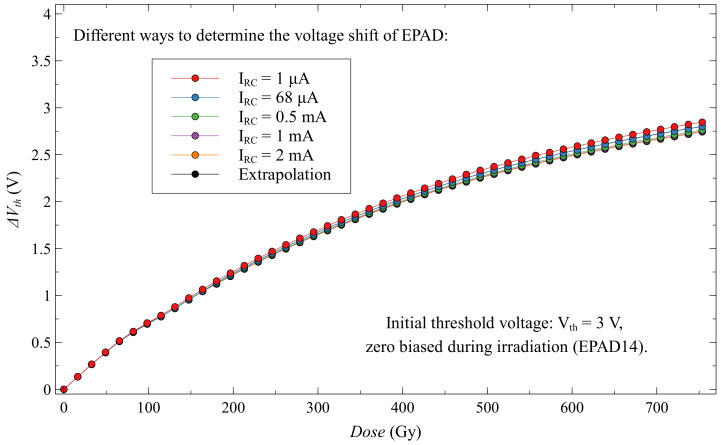
Good example of different Reader-Circuit values.

**Figure A2:**
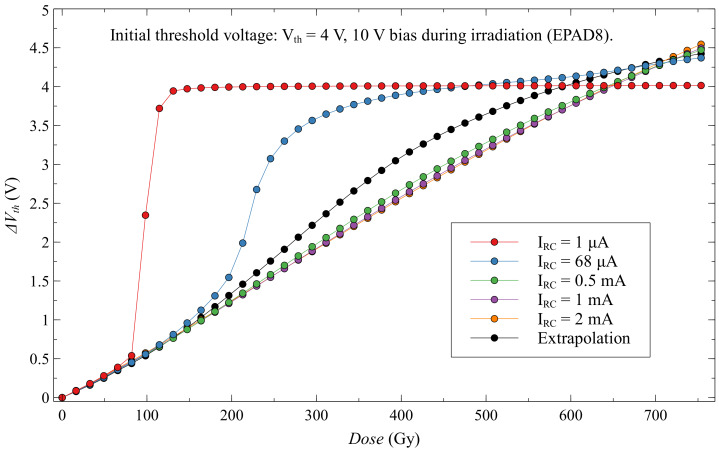
Bad example of different Reader-Circuit values.

**Figure A3:**
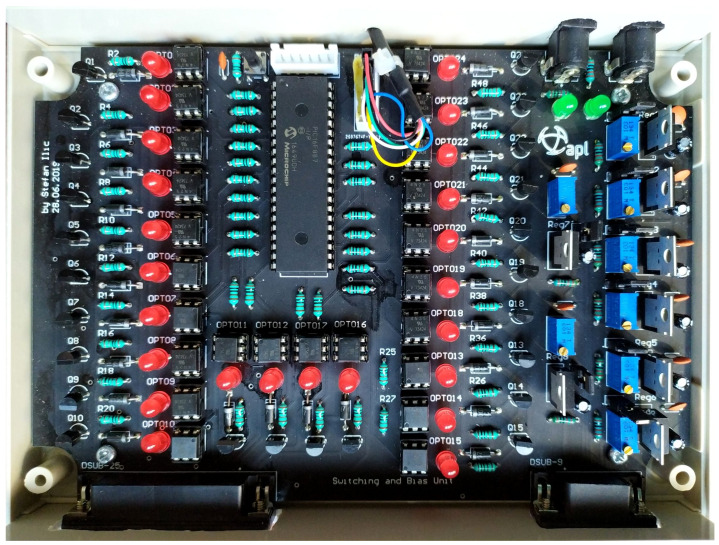
Switching And Bias Unit.

**Figure A4:**
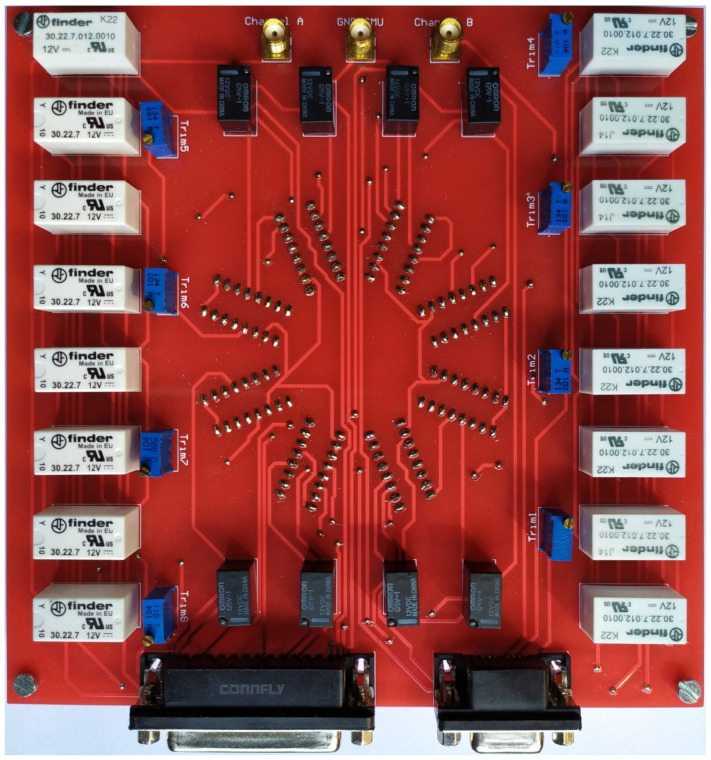
PCB top of mainboard with relays.

**Figure A5:**
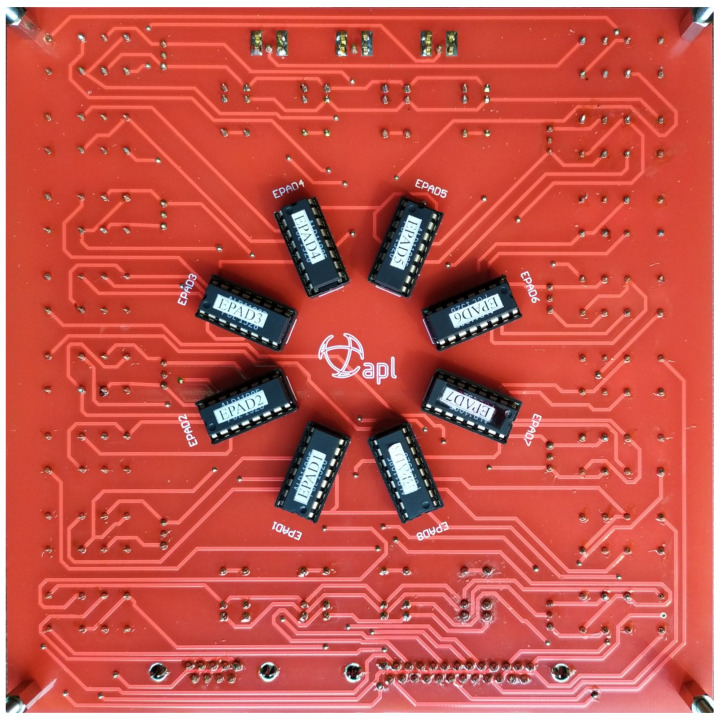
PCB bottom of mainboard with irradiated EPADs.

Because of the above mentioned, it is crucial to wisely choose the reader-circuit current for monitoring the voltage shift. We confirmed that if transfer characteristic is not degraded, there is no difference between results obtained with different reader-circuit currents. We call it a good example and this figure can be seen in Appendix A, Figure A1, we also show the bad example, where the characteristic is degraded (Figure A2). In these figures, it is also shown the extrapolation method which is determined in MATLAB program packet by the transfer characteristics in saturation, as the intersection between $V_{G}$-axis and the extrapolated linear region of the ${\left(I_{D}\right)}^{1/2}-V_{G}$ curves, using the least square method [33].

**Figure 14:**
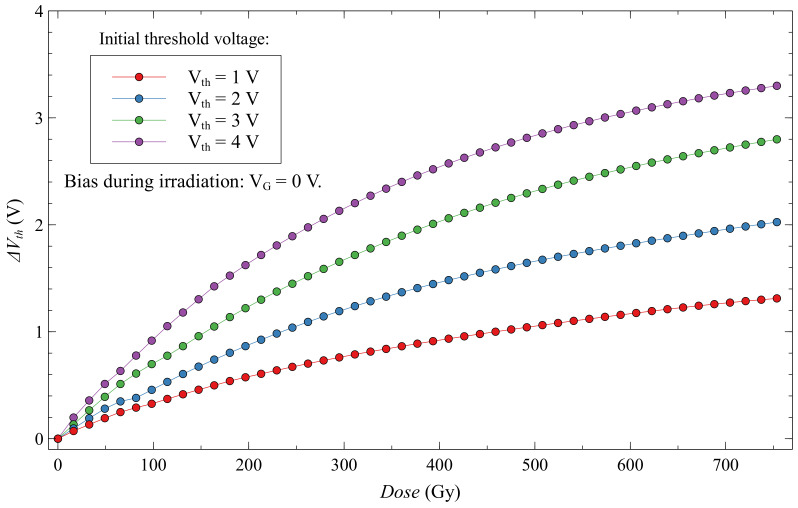
Threshold voltage shift for EPADs with zero bias during irradiation.

**Figure 15:**
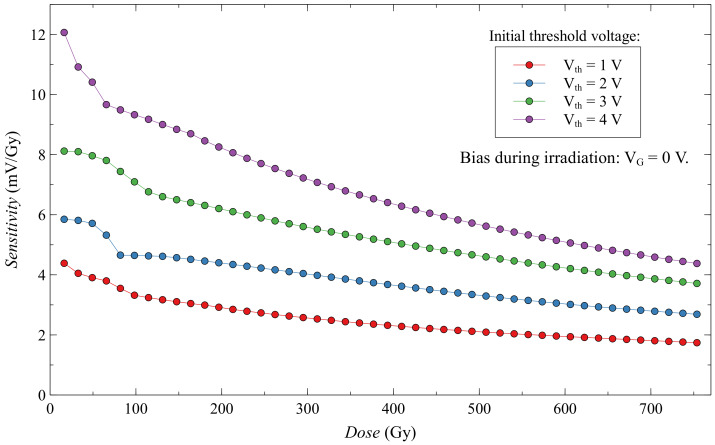
Sensitivity of EPADs with zero bias during irradiation.

Threshold voltage shift for a subgroup of EPADs with zero bias is shown in Figure 14. We can observe a threshold voltage shift depending on the absorbed dose for four different values of the initial threshold voltage. The most significant voltage shift with the absorbed dose has EPAD with the highest initial threshold voltage. Thus, the sensitivity increases with the initial threshold voltage, i.e., with the amount of charge on the floating gate. We note that the dependence of the threshold voltage shift with the dose is not linear, but it decreases, and thus the sensitivity decreases during irradiation. It can be better seen in Figure 15, where is shown the sensitivity of these EPADs with absorbed dose. The sensitivity was calculated as the change in threshold voltage divided by the value of the absorbed dose. As the floating gate is discharging during irradiation, its electric field weakens and, consequently, a smaller generation of electron-hole pairs occurs, resulting in less discharging of the floating gate. For this reason, the sensitivity of EPADs with only an internal electric field (from the FG) decreases with the absorbed dose.

**Figure 16:**
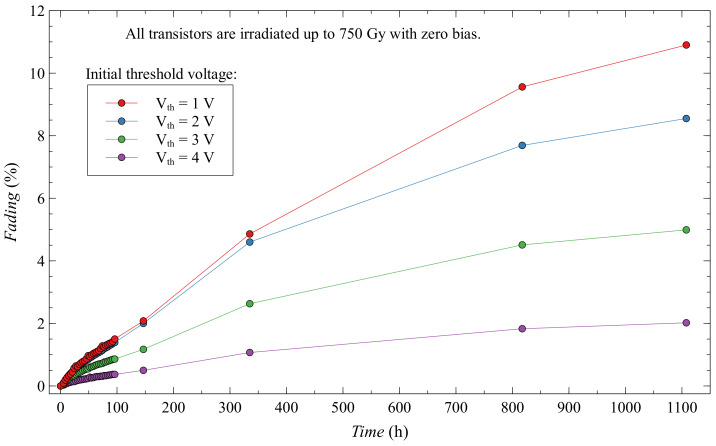
Fading of EPADs with zero bias during irradiation.

The fading represents the deviation percentage of the threshold voltage during the second phase of the experiment, and it was calculated using the following equation [31]:

(1) $$f\left(t\right)=\frac{V_{th}\left(0\right)-V_{th}\left(t\right)}{V_{th}\left(0\right)-V_{th0}},$$

where $V_{th}\left(0\right)$ is the threshold voltage immediately after irradiation, $V_{th}\left(t\right)$ is the threshold voltage after spontaneous recovery, and $V_{th0}$ is the pre-irradiation threshold voltage. Figure 16 shows fading for the first 1100 h after irradiation. We notice that as the initial threshold voltage of the EPAD increases, the fading decreases. We assume that when the electric field from the floating gate is weaker, the holes are more trapped somewhere in the oxide and, after irradiation, create instability.

EPAD with the highest initial threshold voltage, which has the highest sensitivity, also has the lowest fading. This result is in excellent agreement with our previous research on EPAD [27]. After exactly 1107.7 h (about a month and a half), EPAD with zero bias and a 4 V initial threshold voltage has a fading of 2.02%.

**Figure 17:**
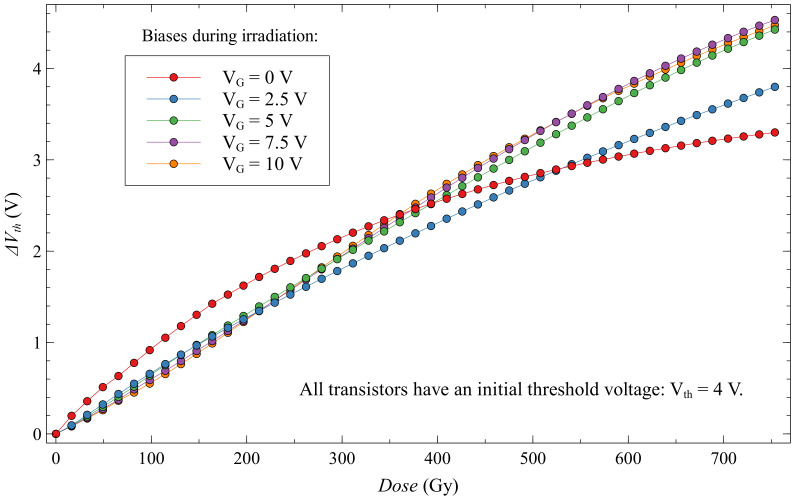
Threshold voltage shift for EPADs with static bias during irradiation.

Looking at EPADs with static bias during irradiation in Figure 17, we can see that they have a more significant voltage shift than EPADs with zero bias. All EPADs have here the same initial threshold voltage, 4 V. Thus, the influence of the external electric field from the control gate helps in the generation of electron-hole pairs during irradiation, as expected. However, during the first 350 Gy, we see that the EPAD with zero bias has a larger shift.

**Figure 18:**
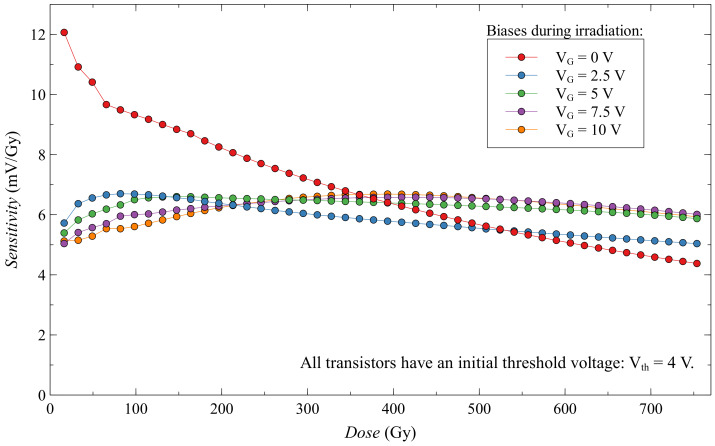
Sensitivity of EPADs with static bias during irradiation.

If we look at the sensitivity of these transistors in Figure 18, we notice that the zero-biased EPAD has a much higher sensitivity at the beginning of the experiment. This phenomenon has already been noted before [27], but we could not explain it at the time. In the discussion, we will try to explain this unusual occurrence in semiconductor dosimeters. We can also note on the same graph that static-biased EPADs have a more linear sensitivity dependence during irradiation compared to zero-biased EPADs, which may be useful in some applications. Still, it should be careful about possible degradation that can occur.

**Figure 19:**
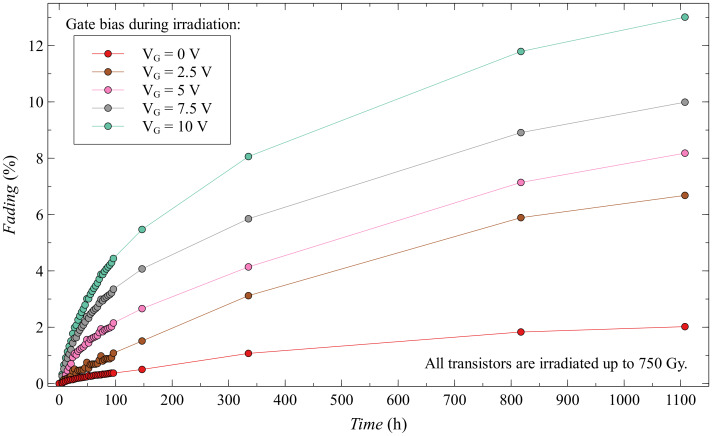
Fading of EPADs with static bias during irradiation.

When looking at fading for this subgroup of EPADs (Figure 19), we can see that again the smallest fading has EPAD with zero bias, and that fading increases with bias. Thus, higher bias does more damage to the EPAD structure, as already demonstrated by the Safe Operation Area.

### 3.2. Dynamic Bias

Looking at the previous section in the Figure 8, we can see that the dynamic bias depends on the two parameters: the supply voltage $V_{DD}$ and the resistor $R_{S}$ in the voltage divider. Assuming that EPAD in a voltage divider is always charged to the same threshold voltage value, in our case it is $V_{th}=4\;V$. The first subgroup in the dynamic group has a voltage value of $V_{DD}=6\;V$ and the second subgroup of $V_{DD}=12\;V$. Depending on the choice of resistor value $R_{S}$, each EPAD is biased in a different voltage range. Information on EPADs is provided in Table 2.

**Figure 20:**
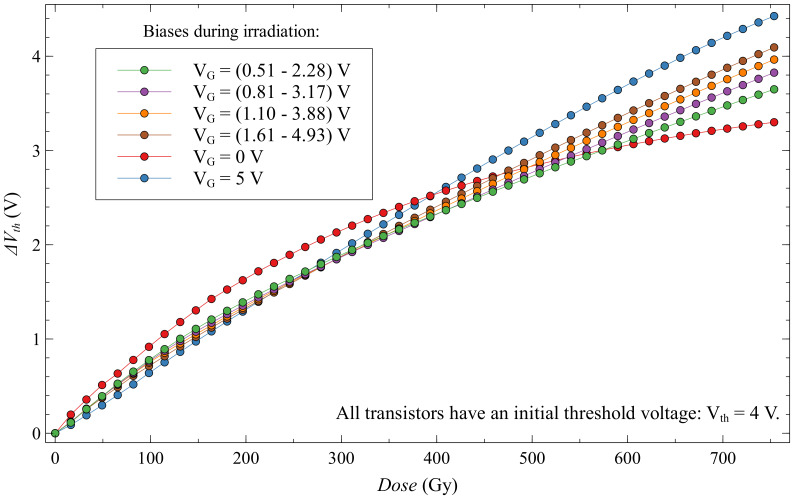
Threshold voltage shift for first subgroup of EPADs with dynamic 6 V bias during irradiation.

Looking at the first subgroup of dynamic biased EPADs compared to EPADs with 0 V and 5 V bias, we can see that the whole subgroup has a very similar dependence of the threshold voltage shift on the absorbed dose (Figure 20). From here we can take EPAD no. 7 with a dynamic range of 1.61–4.93 V as the most linear and with the highest sensitivity. It is also interesting to note that the shift of threshold voltages of this subgroup are distributed between the endpoints of 0 V and 5 V biased EPADs, which corresponds to their dynamic range that they had during irradiation.

**Figure 21:**
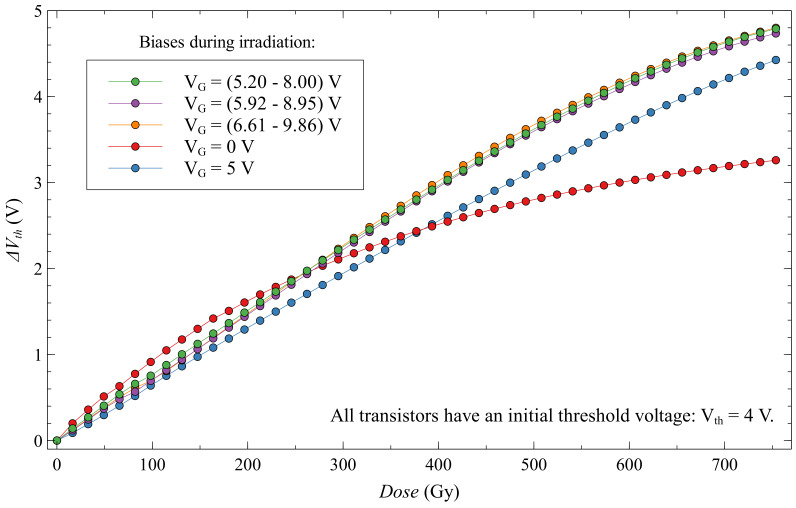
Threshold voltage shift for second subgroup of EPADs with dynamic 12 V bias during irradiation.

The second subgroup of dynamic biased EPADs is shown in Figure 21. Like the first subgroup, they also have very similar dose-dependence changes in threshold voltage. Unfortunately, for one unknown reason, one of the transistors in this subgroup (EPAD no. 15) failed during the experiment, and we will not discuss it further. This subgroup has a higher sensitivity than the first subgroup of dynamic and group of static-biased EPADs. Linearity is also better than for the first subgroup. It is difficult to determine the best in this subgroup because they have very similar characteristics, we consider it to be EPAD no. 9 because it has the closest linear dependence.

**Figure 22:**
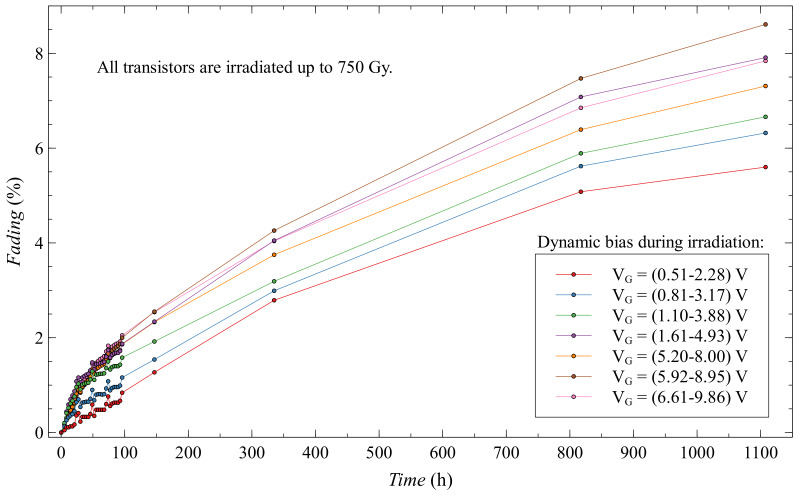
Fading of EPADs with dynamic bias during irradiation.

When it comes to fading for a whole group of dynamic biased EPADs, some distributions can be observed due to different biasing (Figure 22). With the higher values of the dynamic range we have higher fading, as the subgroups themselves have very similar characteristics, it is difficult to draw such a conclusion because there are overlaps. It is important to emphasize that for the whole group of dynamic biased EPADs the value of the fading after 1100 h does not exceed 9%.

## 4. Discussion

In the discussion, we will try to explain the higher sensitivity of zero-biased EPAD compared to static bias group, and to look at the perspective of EPADs and their future applications.

### 4.1. Energy Band Diagram

**Figure 23:**
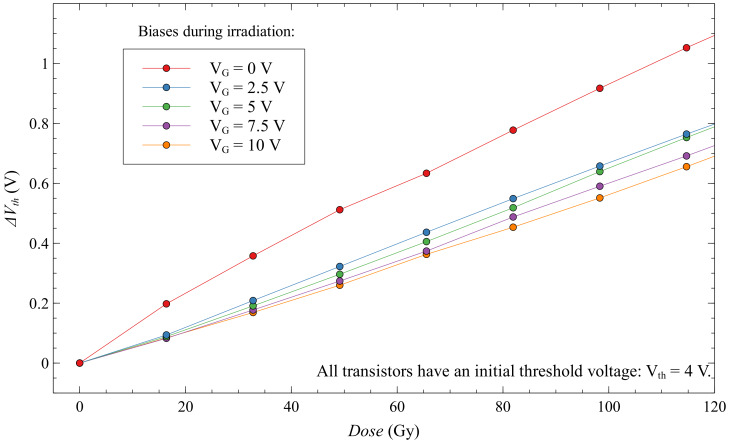
Interesting phenomenon of higher sensitivity with zero-biased floating-gate MOS transistor.

First, we must take a look more closely to the Figure 17 at first 120 Gy of experiment (Figure 23). It can be noticed the distribution of threshold voltage shifts with the control gate bias. Therefore, a higher value of control gate bias produces lower sensitivity in the first part of the experiment.

**Figure 24:**
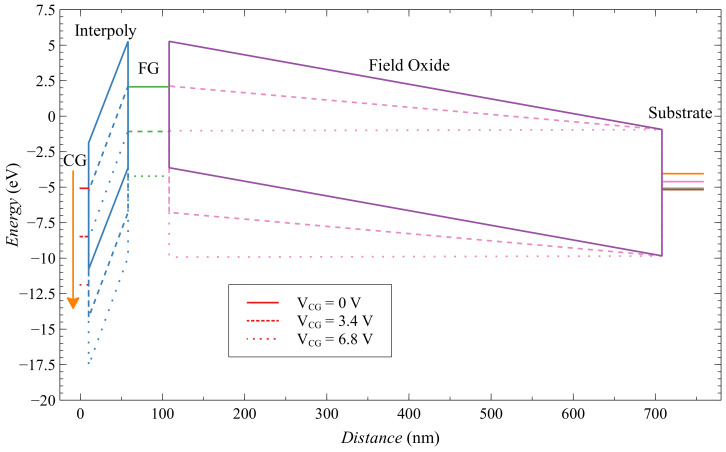
Energy band diagram of EPAD structure.

To explain this very interesting phenomenon, that zero-biased floating-gate MOS transistor has higher sensitivity than static-biased transistors, we must analyze this problem in the perspective of electric fields in the oxides during irradiation, this can be done with the energy band diagram of the floating-gate MOS structure. We used free Multi-Dielectric Energy Band Diagram program from the Boise State University [34,35]. It is crucial to determine which part of the EPAD structure should be analyzed. As we explained before, the large floating gate area that is above the field oxide is the most important for the functionality of the EPAD structure, and thick field oxide provides a large amount of radiation-generated charge when EPAD is used as a radiation sensor. However, because the EPAD is a commercial component, we do not know the dimensions in the cross-section for needs of the energy band diagram input. Therefore, we used information from the very similar structure that was introduced by Tarr et al. [30]. Its structure has field oxide of 600 nm, and interpoly oxide of 48 nm. We assumed that the floating gate is 50 nm thick, and control gate is 10 nm thick, both are n+ polysilicon in the simulation. In the Figure 24 energy band diagram of EPAD structure is represented.

**Figure 25:**
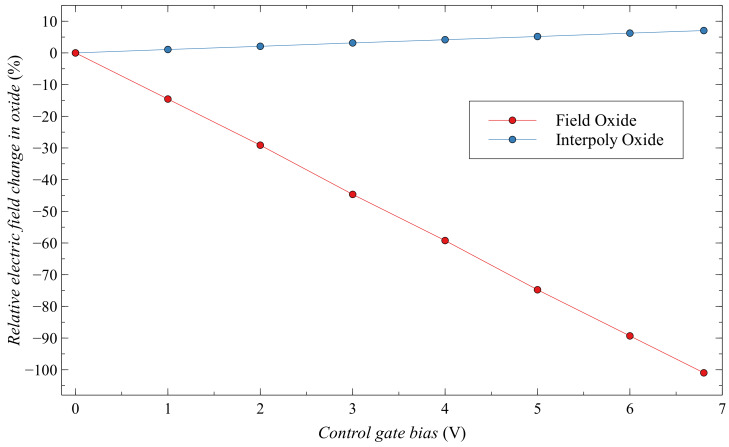
Electric field in field and interpoly oxide.

When we increase the voltage at the control gate, there is a decrease in energy in the floating-gate structure, the electric field in the interpoly oxide and the field oxide changes. Because the field oxide is significantly thicker than the interpoly oxide, changes in the electric field are more drastic. So, we made a graph (Figure 25), where we can see the relative change in the electric field with the change in voltage at the control gate. The electric field strength in field oxide decreases by more than 100% and in interpoly oxide increases by only 7% for 6.8 V bias at the control gate. We can consider that the field change in the interpoly oxide is almost negligible compared to the change in the field oxide. This may explain why biasing the control gate we have a decrease in the sensitivity of EPADs. As the floating-gate decrease, the influence of the control gate bias becomes more dominant.

### 4.2. Epad Future Applications

**Figure 26:**
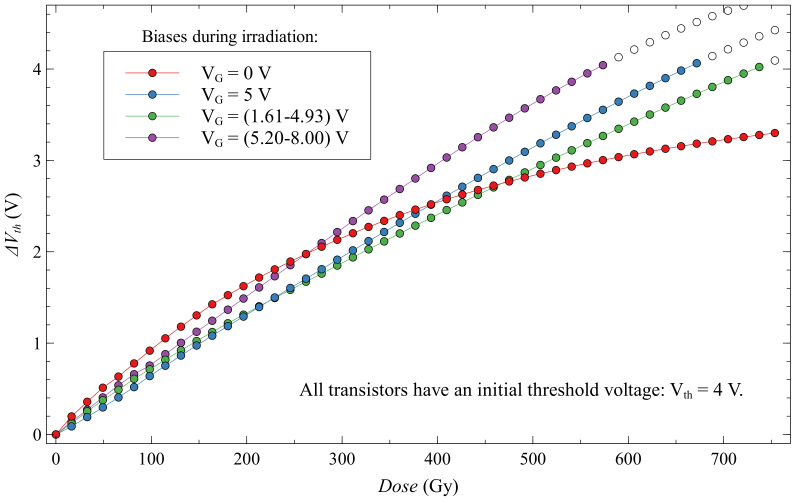
The EPADs with best dosimetric characteristics in all subgroups. White data dots are representing the values after the “four-volt shift”.

From each subgroup in this experiment: zero, static, dynamic 6 V and dynamic 12 V bias, we choose the EPADs with the best characteristics as a radiation sensor (Figure 26). Linearity, sensitivity and stability (fading) were the most important parameters. In this figure, the data of EPADs is shown in “four-volt shift”; after that, white data points are presented, which is because all EPADs are initially charged to 4 V. Therefore, when the threshold voltage is zero, we are considering that the floating gate is almost fully discharged. However, even after that threshold, the transfer characteristic bends, and we can monitor the absorbed dose a little more, but we are not considering that as credible data for the dosimetry application.

The best EPAD in this experiment is EPAD no. 9 with dynamic bias (5.20–8.00) V, it has linear dependence, average sensitivity of 7.55 mV/Gy in 570 Gy dose range and relatively low fading (7.3% after 1100 h). It is promising that some EPADs with dynamic bias have higher sensitivity and better linearity than static-biased EPADs with higher values of biasing. Dynamic biased EPADs may probably have a use that is restricted to the passive application, maybe in space explorations where low power is needed, and the required dose range is appropriate, but the need for sensitivity is not excessively high.

EPAD with zero bias and 4 V initial threshold voltage has definitely the highest sensitivity in the range of 300 Gy, and the lowest fading. However, the dependence of absorbed dose is not linear. Maybe, if we use this biasing configuration in recharging mode, such as presented by the group of authors [36,37,38], the dependence of the absorbed dose can be linear and even with higher sensitivity. In that case, the radiation sensor should have some extended electronics that will continuously measure and do recharging.

## 5. Conclusions

Electrically Programmable Analog Device is a commercial NMOS floating-gate transistor designed for totally different purposes, this paper describes the possibility of using that transistor as a radiation sensor. An experiment was performed at the “Vinča” Institute of Nuclear Sciences with the gamma radiation source. Highlighting the main results, we concluded that a higher bias does more damage to the EPAD structure. EPAD with zero bias and 4 V initial threshold voltage shows the lowest fading and the highest sensitivity in the 300 Gy dose range. The interesting phenomenon that zero-biased floating-gate MOS transistor has higher sensitivity compared to static-biased transistors was explained with the energy band diagram. The idea of the dynamic bias of the control gate during irradiation was presented for the first time. This dynamic method achieved very good results, showing that some EPADs with dynamic bias have higher sensitivity and better linearity than static-biased EPADs with higher values of biasing. Further work is needed on its implementation. Due to the degradation of the transfer characteristics of EPAD during irradiation, a function of the safe operation area has been found that determines the maximum voltage at the control gate for the desired dose, which will not lead to degradation of the transistor. The dosimetric characteristics of EPAD, such as sensitivity, linearity, fading, are promising, but some changes in design need to be made to make this component to operate as a radiation sensor.

## Figures and Tables

**Table 1:** EPADs with static bias during irradiation.

Name	Bias During Irradiation	Initial $V_{th}$
EPAD2	2.5 V	$V_{th}=$ 4 V
EPAD4	5 V	$V_{th}=$ 4 V
EPAD6	7.5 V	$V_{th}=$ 4 V
EPAD8	10 V	$V_{th}=$ 4 V
EPAD10	0 V	$V_{th}=$ 1 V
EPAD12	0 V	$V_{th}=$ 2 V
EPAD14	0 V	$V_{th}=$ 3 V
EPAD16	0 V	$V_{th}=$ 4 V

**Table 2:** EPADs with dynamic bias during irradiation.

Name	$V_{DD}$	$R_{S}$	Biasing
EPAD1	6 V	1 kΩ	(0.51–2.28) V
EPAD3	6 V	2.5 kΩ	(0.81–3.17) V
EPAD5	6 V	6 kΩ	(1.10–3.88) V
EPAD7	6 V	50 kΩ	(1.61–4.93) V
EPAD9	12 V	3 kΩ	(5.20–8.00) V
EPAD11	12 V	6 kΩ	(5.92–8.95) V
EPAD13	12 V	15 kΩ	(6.61–9.86) V
EPAD15	12 V	100 kΩ	(7.40–10.9) V

## Abbreviations

The following abbreviations are used in this manuscript:

EPAD	Electrically Programmable Analog Device
ZTC	Zero Temperature Coefficient
SMU	Source Measure Unit
GPIB	General-Purpose Interface Bus
ICSP	In-Circuit Serial Programming
FTDI	Future Technology Devices International
PMMA	Poly(Methyl Methacrylate)

## Appendix A. Reader-Circuit Currents

Different reader-circuit currents with good and bad example.

## Appendix B. Photos of the Automated Measurement System

Photos of SABU and mainboard with irradiated EPADs.
